# Gasdermin D in peripheral nerves: the pyroptotic microenvironment inhibits nerve regeneration

**DOI:** 10.1038/s41420-021-00529-6

**Published:** 2021-06-14

**Authors:** Ye Tao, Fang Wang, Zhaohui Xu, Xianfu Lu, Yanqing Yang, Jing Wu, Changyu Yao, Fangzheng Yi, Jiajia Li, Zhigang Huang, Yehai Liu

**Affiliations:** 1 Department of Otolaryngology-Head and Neck Surgery, The First Affiliated Hospital of Anhui Medical University, Hefei, 230022 China; 2 Department of Neurology, The First Affiliated Hospital of Anhui Medical University, Hefei, 230022 China; 3 Department of Disease prevention and control, Xijing 986 Hospital, The Fourth Military Medical University, Shanxi, 710000 China; 4 Department of Anesthesiology (High-Tech Branch), The First Affiliated Hospital of Anhui Medical University, Hefei, 230080 China; 5 Department of Clinical Laboratory, The First Affiliated Hospital of Bengbu Medical College, Bengbu, Anhui 233000 China; 6 The Center for Scientific Research of the First Affiliated Hospital of Anhui Medical University, Hefei, 230022 China; 7 Department of Otolaryngology-Head and Neck Surgery, Key Laboratory of Otolaryngology-Head and Neck Surgery, Beijing Tongren Hospital, Capital Medical University, Beijing, 100730 China

**Keywords:** Cell death in the nervous system, Innate immunity

## Abstract

Wallerian degeneration (WD) involves the recruitment of macrophages for debris clearance and nerve regeneration, and the cause of the foamy macrophages that are frequently observed in peripheral transection injuries is unknown. Recent studies indicated that these foamy cells are generated by gasdermin D (GSDMD) via membrane perforation. However, whether these foamy cells are pyroptotic macrophages and whether their cell death elicits immunogenicity in peripheral nerve regeneration (PNR) remain unknown. Therefore, we used GSDMD-deficient mice and mice with deficiencies in other canonical inflammasomes to establish a C57BL/6 J mouse model of sciatic nerve transection and microanastomosis (SNTM) and evaluate the role of GSDMD-executed pyroptosis in PNR. In our study, the *GSDMD*^−*/*−^ mice with SNTM showed a significantly diminished number of foamy cells, better axon regeneration, and a favorable functional recovery, whereas irregular axons or gaps in the fibers were found in the wild-type (WT) mice with SNTM. Furthermore, GSDMD activation in the SNTM model was dependent on the NLRP3 inflammasome and caspase-1 activation, and GSDMD-executed pyroptosis resulted in a proinflammatory environment that polarized monocytes/macrophages toward the M1 (detrimental) but not the M2 (beneficial) phenotype. In contrast, depletion of GSDMD reversed the proinflammatory microenvironment and facilitated M2 polarization. Our results suggested that inhibition of GSDMD may be a potential treatment option to promote PNR.

## Introduction

Peripheral nerve injuries (PNIs), particularly transection injuries, have resulted in extensive disability worldwide [1]. Although microanastomosis can be used to connect the distal and proximal nerves, the clinical outcomes of peripheral nerve regeneration (PNR) remain unsatisfactory and therefore require further exploration [2].

PNR involves neuron degeneration, in which injured axons and their surrounding myelin-sheathes undergo the dying-back process or retrograde degeneration, termed Wallerian degeneration (WD) [3]. WD can produce high levels of myelin and axon debris and other damage-associated molecular patterns (DAMPs), thereby initiating innate immune responses and promoting macrophage infiltration [4]. Furthermore, Schwann cells and infiltrated macrophages coordinate and orchestrate efficient PNR procedures, including macrophage polarization (M1, inflammation-promoting; M2, repair-enhancing), clearance of myelin debris, Büngner band formation, neurotrophic factor secretion, and axon regeneration [5–7]. However, functional recovery is unfavorable when the injury results in excess inflammation [6].

As a known canonical inflammasome, the multiprotein NOD-, LRR-, and pyrin domain-containing protein 3 (NLRP3) inflammasome complex can result in caspase-1 cleavage [8, 9], which specifically cleaves gasdermin D (GSDMD) between the amino-terminal gasdermin-N (GSDMD-N) and carboxy-terminal gasdermin-C domains (GSDMD-C); this process is essential and sufficient for membrane pore formation and a robust proinflammatory death process termed pyroptosis [10]. Specifically, pyroptotic macrophages release many proinflammatory cytokines, other DAMPs (Ca^2+^, ATP, and other cytoplasmic contents and cell corpses), thereby amplifying the cascade of immune responses. Caspase-1 inhibition could attenuate neural damage and improve neurobehavioral performance in the central nervous system (CNS) [11]. In peripheral transection injuries, the role of macrophage pyroptosis in PNR has been fully explored, but the cause of foamy macrophage accumulation is unclear [12].

Therefore, the objectives of this study were as follows: (1) generate a C57BL/6 J mouse model of sciatic nerve transection and microanastomosis (SNTM); (2) compare the foamy phenomenon of pyroptosis and the functional recovery of PNR between GSDMD^−/−^ and WT mice with SNTM; and (3) determine whether GSDMD cleavage is dependent on the inflammasome and its impact on macrophage polarization and the cytokine environment.

## Methods

### Ethics statement and Mice

The ScN showed an equivalent capacity for PNR in nonhuman primates and C57BL/6 J mice [13], in which rapid WD that reflected the natural WD progression was identified [14]. C57BL/6 J mice were obtained from the Model Animal Research Center of Nanjing University. *NLRP3*^*−/*^^−^, *ASC*^*−/*−^, *AIM2*^*−/−*^, *IL-1β*^*−/*−^, and *caspase-1*^*−/−*^ mice were described previously [15], and *GSDMD*^*−/*−^ and *NLRC4*^*−/*−^ mice were purchased from GemPharmatech Co., Ltd. Since testosterone can upregulate neuritin mRNA expression and thereby enhance PNR [16], we only adopted female mice in this study to exclude the effects of testosterone. We used specific pathogen-free (SPF) conditions for mouse feeding and maintenance under a strict 12-hour light cycle (lights on at 8 a.m. and off at 8 p.m.). The Animal Care Committees of the University of Science and Technology of China and Anhui Medical University approved all experimental animal protocols. Although we try to minimize the mice number and maximize the use of mice tissues, a total of 69 mice was used in this study. The detailed information was as follows: each group of WT and *GSDMD*^−*/−*^ contained six mice, and each group of *NLRP3*^*−/*−^, *ASC*^*−/−*^, *AIM2*^*−/*−^, *IL-1β*^−*/−*^, *caspase-1*^−*/−*^, and *NLRC4*^*−/−*^ contained three mice for SNTM model and sciatic function evaluation, ScN electrophysiological testing and pathological section assessment (*n* = 30); furthermore, each group of WT and *NLRP3*^*−/*−^ and *GSDMD*^*−/*−^ contained three mice for transmission electron microscopy study (*n* = 9); additionally, each group of WT and *GSDMD*^*−/−*^ contained fifteen mice for flow cytometry and mRNA expression assessment and western blotting (*n* = 30). In addition, no blinding method was applied in this study.

### Model of SNTM

We established a mouse model of SNTM using the following procedures: (1) anesthesia was administered by face mask inhalation of 5% halothane, the thigh hair was removed, and the skin was sterilized; (2) a horizontal skin incision along the femur was made to expose the biceps femoris (BF) muscle; (3) the BF was pulled down, and the ScN was exposed as previously described [17], and we directly made an ScN transverse injury via a vertical sharp-cut; and (4) we used the epineurial repair technique for end-to-end anastomosis with a 12–0 nylon suture under a stereomicroscope (Olympus, SZ61). The detailed procedures for microanastomosis are shown in Supplemental Fig. 1. Specifically, the mice inclusion and exclusion criteria was as follows: inclusion criteria, successful SNTM within 10 min (long time operation can cause ScN swelling and its content disclosure); exclusion criteria, SNTM surgery failed to connect the distal and proximal segment of ScN, polluted surgery cites with potential infection risk and surgery time more than 10 min.

### Assessment of ScN functional index (SFI)

We used walking track analysis (ink track method) for the assessment of the SFI, in which parameters of the ink track method were collected as follows: print length (PL, the distance between the heel and the third toe), toe spread (TS, the distance between the first and fifth toes), and intermediate toe spread (ITS, distance between the second and fourth toes). With these collected parameters (TS, ITS, and PL), the SFI was calculated with the following formula [18]: (1) toe spread factor (TSF) = (TS experimental − TS normal)/TS normal and print length factor (PLF) = (PL experimental − PL normal)/PL normal; (2) SFI = 118.9 × TSF − 51.2 × PLF − 7.5. Furthermore, the SFI value ranged from −100 (transected ScN) to 0 (sham operation); therefore, a smaller SFI indicated a worse ScN function [18].

### Motor nerve conduction velocities (MNCVs)

We used the Medelec Sapphire II electromyography unit to stimulate and record the compound motor action potentials (CMAPs) and the MNCVs, measured in the ScN. Mice were anesthetized by face mask inhalation of 5% halothane and fixed on a tablet. After disinfection of the proximal and distal ends of the lower limb with 75% alcohol, we used two bipolar electrodes for stimulation: one placed on the ScN proximally near the obturator foramen and the other on the common peroneal nerve distal to the division. This method of electrode placement resulted in the maximal distance between them. In addition, the electrode stimulation was set at a constant current of 50 μsec as previously described [19]. MCV was calculated by the conventional method: MCV (m/sec) = L/T, in which L (m) is the distance between the two stimulus electrodes and T (sec) is the difference in delay between CMAPs evoked by the proximal and distal stimulating electrodes.

### cDNA library preparation and qPCR analysis

Total RNAs were isolated with Trizol reagent (15596026, ThermoFisher) and were transcribed reversely to cDNAs with a Prime-Script reagent kit (RR037B, TaKaRa). We used cDNAs SYBR Green Premix Ex Taq (RR820B, TaKaRa) to amplify cDNAs via Applied Biosystems Stepone real-time PCR System (Applied Biosystems, Foster City, CA, United States). The primer sequences are listed in Supplemental Table 1. The comparative CT (ΔΔCT) method was used to quantify target expression with normalization to the housekeeping gene GAPDH.

### Western blotting

ScN segments of the SNTM bridge (2 mm length) were dissected precisely under a stereomicroscope (Olympus, SZ61) and then subjected to protein extraction. We used direct homogenization and laemmli buffer to isolate and lyse protein samples. We used 25-gauge needles to aspirate protein lysates and subsequently centrifuged the aspiration at 14,000 rpm for 10 min.

We used β-mercaptoethanol, glycerin, and bromophenol-blue to mix and dissolute the collected supernatants and incubated at 95 °C for 5 min. Subsequently, equal amounts of protein samples were resolved in 10% SDS-PAGE and electrotransferred to polyvinylidene fluoride (PVDF) membranes. We blocked these membranes in 5% nonfat dry milk for 2 h at room temperature and probed with primary antibodies: anti-Pro-Caspase-1 (1:1000; Abcam, ab179515), anti-Caspase-1 (1:1000; Invitrogen, PA5-99390), or anti-GSDMD (1:400, Abcam, ab219800), or anti-β actin (1:2000; ab8226, Abcam) at 4 °C overnight. Subsequently, the membranes with primary antibodies were incubated with HRP-conjugated secondary antibodies (1:5000; Pierce, Rockford, IL, United States) for 1 h at room temperature and then developed with enhanced chemiluminescence reagent (Pierce). The quantification of band intensity was performed by using Bio-Rad ChemiDoc XRS + System.

### Pathological section and double fluorescent immunohistochemistry (IHC) staining

Mice were rapidly euthanized via a high concentration of carbon dioxide, and we obtained nerves with both proximal and distal parts, ~10–15 mm in length; furthermore, the harvested ScNs underwent paraformaldehyde fixation and paraffin embedding, and embedded tissues were cut into 5 μm thick sections by a rotary slicer (LEICA RM2135, Wetzlar, Germany). According to the staining protocols, we used hematoxylin and eosin (H&E) staining and silver staining for conventional and nerve axon observation according to the staining protocols, respectively [20]. The H&E staining procedures were as follows: the paraffin sections (in staining racks) were dewaxed in xylene and hydrated in a gradually decreasing series of ethanol (100% ethanol for 3 min, 95% ethanol for 3 min, 80% ethanol for 3 min, 70% ethanol for 3 min, and distilled water for 10 min); the sections were stained in hematoxylin solution for 3 min and placed under running tap water at room temperature for at least 5 min and subsequently stained in working eosin Y solution for 2 min; the stained sections dehydration also undergone a series of ethanol and xylene (dip the slides in 95% ethanol about 20 times, 95% ethanol [2 min, 2 times, 2 × 2], 100% ethanol [2 min, 2 times, 2 × 2], xylene [2 min, 3 times, 2 × 3]). Similarly, the silver staining method was similar to the H&E staining, the Silver nitrate (20%) solution was heated at 60 °C for 15 min, and then dewaxed sections were placed into the solution for 15 min and rinsed with tap water.

The double fluorescent IHC staining incubation procedures were as follows: 1% BSA in PBST for 30 min to block unspecific binding of the antibodies; the mixture of two primary antibodies (anti-F4/80, 1:400 and anti-iNOS, 1:200; or anti-F4/80, 1:400 and anti-CD206, 1:200) in 1% BSA in PBST in a humidified chamber for 1 h at room temperature; the mixture of two secondary antibodies which are raised in different species in 1% BSA for 1 h at room temperature in the dark; 1 μg/ml DAPI (DNA stain) for 1 min. After incubation and washing with TBST (5 min, 3 times, 5 × 3), we mount the coverslip with an anti-fading medium and observe with a confocal microscope.

### Processing samples for transmission electron microscopy

We precisely harvested nerve samples located at the distal segment and the bridge of the end-to-end anastomosis under a stereomicroscope (Olympus, SZ61), and the sample was ~0.5–1 mm in diameter. Consequently, the sample underwent a series of complex procedures: fixation, postfixation, dehydration, block staining, embedding, and preparation of ultrathin sections (Supplemental table 2.) [21, 22]. We used an ultrathin microtome (Leica, U7) for ultrathin section cutting and a 120 kV transmission electron microscope (Tecnai G [23] Spirit BioTWIN, FP5018/41) for imaging acquisition.

### Flow cytometry

Mice that underwent SNTM were euthanized at 7 and 14 days after repair to assess the early and middle phases of the immune responses to PNI. The regenerative bridge and its distal ScN segment (~10 mm length) were cut and placed in a petri dish with 1 mL of RPMI-1640 (Corning) and then cut into 1 mm pieces. Then, the tissues were transferred to a 50-mL conical tube with 10 mL of digestion buffer (comprised of 3 mg/mL collagenase type I [Sigma, C1-22], 1 mg/mL hyaluronidase [Sigma, 37326-33-3], and 0.5 mL of 1 mM HEPES in RPMI-1640). The digestion was performed for 1 h in a 37 °C shaker, and we obtained a single-cell suspension via a 70-μm mesh strainer (BD Biosciences). RBC blood cell lysis buffer (00-4333-57) was used for RBC lysis, and cells were incubated with membrane antibodies (anti-CD45, anti-CD11b, anti-F4/80). Subsequently, washed cells were fixed and permeabilized with Cytofix/Cytoperm (BD Biosciences) for 20 min at 4 °C. Once fixed, cells were incubated with intracellular antibodies. The excluded antibody markers were Ly6G (granulocytes), while the positive selection markers for macrophages included CD45 (total lymphocytes), CD11b (bone marrow-derived monocytes), F4/80 (macrophages), iNOS (M1), and CD206 (M2). The gating strategy was shown in Supplemental Fig. 2.

### Antibodies and reagents

The anti-mouse antibodies were as follows: anti-procaspase 1 antibody (Abcam, ab179515), anti-cleaved caspase 1 antibody (Invitrogen, PA5-99390), anti-GSDMD antibody (Abcam, ab219800), anti-GSDMD-N (Abcam, ab219800), anti-F4/80 (Abcam, ab16911), anti-iNOS antibody (Abcam, ab3523), anti-CD206 antibody (Invitrogen, MA5-16871; Abcam, ab64693), anti-β actin (Abcam, 8226), V450-conjugated Ly6G (BD, 560453), V500-conjugated CD45 antibody (BD, 562129), PerCP5.5-conjugated CD11b (Invitrogen, 45-0112-82), APC-conjugated F4/80 (eBioscience, 17-4801-82), PE-cy7-conjugated iNOS (Invitrogen, 25-5920-82), and PE-conjugated CD206 (eBioscience, 25-5920-82).

### Statistical analysis

We used SPSS (IBM SPSS software, version 22.0) for statistical analysis and Image J (ij153-win-java8) for double fluorescent positive cell counting. Unpaired Student’s *t*-tests (two-sided) were used for subgroup comparisons.

## Results

### Macrophage pyroptosis occurred during SNTM

**Fig. 1:**
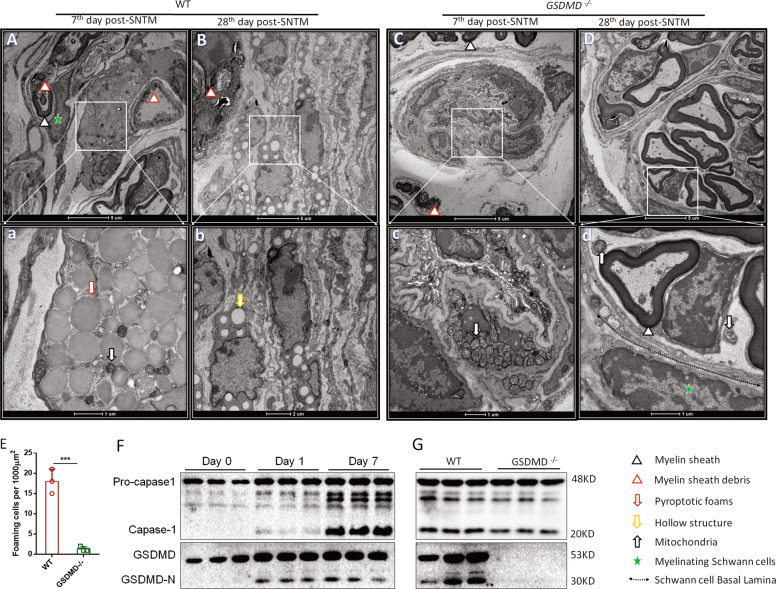
GSDMD deficiency resulted in reduced pyroptotic foaming cells and decreased caspase-1 in the SNTM bridge. **A** In the bridge of the WT SNTM group, foaming macrophages underwent pyroptosis near the degenerating myelin sheath, and several enlarged swollen mitochondria were found near these foams (a) on the 7th day post-surgery. **B** On the 28th day, these pyroptotic foams turned into vacuole structures after releasing their inflammatory contents, the vacuole structures (b) were near the remnant nuclei, and they were surrounded by sheath debris and fibrotic and necrotic structures, whereas few regenerated nerve sheaths and axons were found. **C** In the bridge of the *GSDMD*^−*/*−^ SNTM group, several infiltrating macrophages were found near the degenerating myelin sheath. These macrophages died without foaming. Many swollen mitochondria were found near the remnant nuclei. (c) On the 7th day post-surgery. **D** On the 28th day, the *GSDMD*^*−/*−^ SNTM group showed regenerated myelin sheaths and axons, and depolarized Schwann cells had formed the Büngner band, where a few dead mitochondria were found. **E** *GSDMD*^−*/*−^mice demonstrated significantly decreased foaming cells in the SNTM bridge, compared to their WT control (***p < 0.0001; *n* = 3). **F** The dynamic expression of caspase-1 and GSDMD-N (cleaved by pro-caspase-1 and GSDMD, respectively) in the WT SNTM (on day 0, day 1, and day 7) suggested the occurrence of GSDMD-N executed pyroptosis, with particular significance on the 7th day (*n* = 3). **G** *GSDMD*^*−/−*^ SNTM had decreased cleaved caspase-1 expression and deficiency of GSDMD and GSDMD-N expression, compared to their WT SNTM on the 7th day post-operation (*n* = 3).

To determine whether macrophage pyroptosis plays a role in PNR, we first used electron transmission microscopy to visualize foamy macrophages during SNTM. As shown in Fig. 1A, the foamy cells disrupted the integrity of the cell membrane and released other disintegrated intracellular organelles, which is a typical pyroptotic phenomenon that was observed in the bridge (distant segment) of the WT mice with SNTM on the 7th day. Furthermore, on the 28th day, these pyroptotic foams turned into hollow bubbles after releasing their inflammatory contents, and these hollow bubbles were observed near the remnant nuclei of pyroptotic cells. These pyroptotic macrophages were mainly surrounded by sheath debris, fibrosis, and necrotic structures, with few regenerated nerve sheaths and axons (Fig. 1B). In contrast, the GSDMD^−/−^ mice with SNTM did not demonstrate a similar foaming phenomenon on the 7th day and displayed well-regenerated myelin debris and axons on the 28th day (Fig. 1C, D). Therefore, we counted the number of pyroptotic foaming cells and found a significant difference between the WT and GSDMD^−/−^ mice (Fig. 1E). These results indicated that GSDMD induced pyroptosis in the WT SNTM group with an amplified inflammatory cascade that inhibited the PNR.

Inflammasome-dependent caspase-1 activation was reported to act upstream of GSDMD cleavage and subsequent pyroptosis. Therefore, we assessed the activation of caspase-1 and GSDMD in the SNTM bridge (distant segment) in the WT and GSDMD^−/−^ mice and found that SNTM could indeed induce cleaved caspase-1 and GSDMD (Fig. 1F, G). This finding indicated that GSDMD-dependent macrophage pyroptosis occurred during SNTM.

### GSDMD deficiency resulted in improved PNR in the SNTM model

**Fig. 2:**
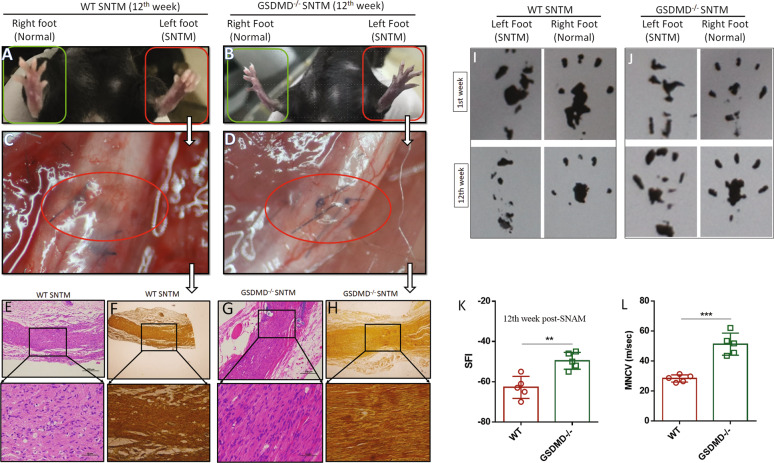
The GSDMD-deficient group demonstrated a better PNR than the WT group in the SNTM model. **A**, **B** show the comparisons of the foot states between the SNTM group (left) and the normal control group (right) at the 12th week, in which the WT SNTM foot was curled up, while the *GSDMD*^−*/*−^ mouse showed a stretched food similar to that of the normal control. **C**, **D** show WT and *GSDMD*^−*/*−^ SNTM bridge vascularization, in which the regenerated microvasculature in the WT mice was irregular and spreading, while vascularization in the *GSDMD*^*−/*−^ mice demonstrated clear microvascular regeneration. **E**, **F** H&E and silver staining showed the disorganized nuclear arrangement and irregular axon courses in the WT SNTM bridge. **G**, **H** The *GSDMD*^*−/−*^ SNTM bridge had a parallel organization of both the cell nuclear arrangement and the course of the regenerated axons, as shown by H&E and silver staining. **I** Footprint demonstration of the WT mice with SNTM in the 1st week and 12th week. The WT mice with SNTM showed inhibited PNR and a curled footprint, while **J** an effectively stretched footprint was observed in the GSDMD^−/−^ mice with SNTM after 12 weeks of recovery. **K**, **L** show the SFI and MNCV comparisons between the WT and *GSDMD*^−*/*−^ mice, and significant differences were found (*n* = 6). *p < 0.05; **p < 0.01; ***p < 0.001; ****p < 0.0001.

Since pyroptosis could trigger the innate immune response, which may dampen the PNR, we used the SNTM mouse model to compare the morphological differences and evaluate ScN function between the WT and GSDMD^−/−^ mice. In this comparison, the foot of the WT mice was curled (Fig. 2A), while a stretched foot was observed in the GSDMD^−/−^ mice after a 12-week recovery (Fig. 2B). Importantly, vascularization plays a significant role in effective PNR by directing Schwann cell migration [24]. Therefore, we observed the vascularization of the SNTM bridge in vivo and found that the regenerated microvasculature in the WT mice was irregular and spreading (Fig. 2C), while that in the GSDMD^−/−^ mice demonstrated clear microvascular regeneration (Fig. 2D). Furthermore, H&E and silver staining showed a disorganized nuclear arrangement and irregular axon courses in the WT SNTM bridge (Fig. 2E, F), while the GSDMD^−/−^ SNTM bridge had a parallel organization of both the cell nuclear arrangement and the course of the regenerated axons (Fig. 2G, H). We also assessed the footprints of the SNTM-treated mice and found that the GSDMD^−/−^ mice had a better foot shape than the WT mice (Fig. 2I, J). Consistently, comparisons of both the SFI and MNCVs between the WT and GSDMD^−/−^ mice with SNTM showed significant differences (*p* < 0.05; Fig. 2K, L). Taken together, these results demonstrated that GSDMD deficiency facilitates the PNR in the SNTM mouse model.

### NLRP3 inflammasome-dependent pyroptosis determined PNR

**Fig. 3:**
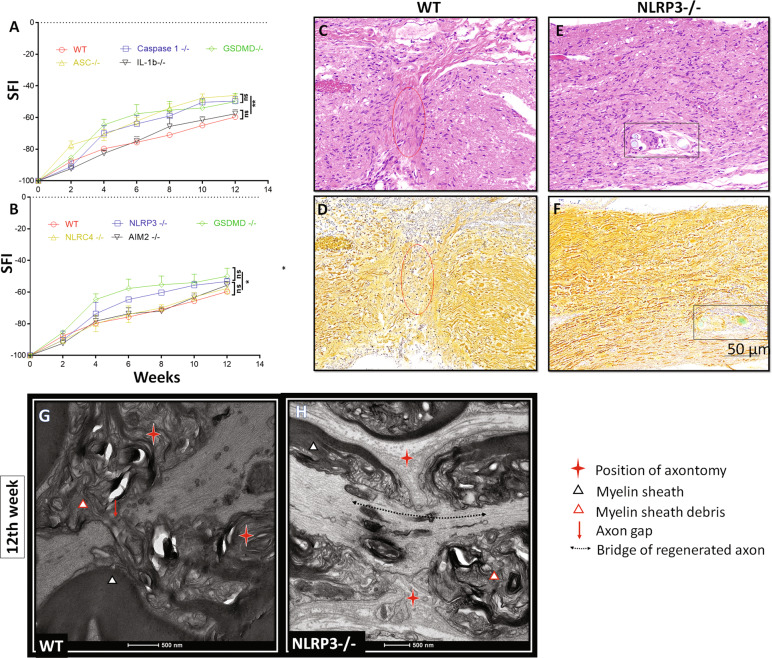
The NLRP3 inflammasome rather than other inflammasomes or IL-1β determined the PNR effects. **A** The *GSDMD*^−/−^, *ASC*^*−/*−^, and *caspase-1*^*−/*−^ mice had better recovery of the SFI than the WT and *IL-1β*^−*/−*^ mice (*n* = 3). **B** The *NLRP3*^−*/*−^ mice had better recovery of the SFI than the *NLRC4*^−*/*−^ and *AIM2*^−*/*−^ mice; furthermore, the *NLRP3*^*−/−*^ mice had an SFI similar to that of the *GSDMD*^−*/−*^ mice (*n* = 3). **C**, **D** H&E and silver staining of a bridge of the WT SNTM group, in which a gap was found between the proximal and distant nerve stumps. This gap (red oval area), attributed to fibrosis, hampered axon regeneration and resulted in an unfavorable PNR. **E**, **F** H&E and silver staining of a bridge of the NLRP3^−/−^ SNTM group, in which both regenerated axons and their supporting cell nuclei had a parallel arrangement and favorable PNR. In addition, the rectangular area shows the residual sutures. **G**, **H** Transmission electron microscopy of longitudinal sections of the WT and *NLRP3*^*−/−*^ mice with SNTM. In the WT SNTM group, transmission electron microscopy also identified a gap (red arrow) between the proximal and distant nerve stumps, and this gap hampered axon regeneration, while a bridge of reconnected axons was found in the *NLRP3*^−*/−*^ SNTM group; the black dashed line indicates the course of axons. *p < 0.05; **p < 0.01; ***p < 0.001; ****p < 0.0001.

Macrophages express many pattern recognition receptors (PRRs), which may recognize the DAMPs generated during PNI to initiate inflammasome assembly and thereby trigger pyroptosis [10]. To confirm that inflammasome-dependent GSDMD activation and pyroptosis prevent PNR, we further used the SNTM model in ASC^−/−^, caspase-1^−/−^, and IL-1β^−/−^ mice and found that the GSDMD^−/−^, ASC^−/−^, and caspase-1^−/−^ mice had better recovery of the SFI than the WT and IL-1β^−/−^ mice (Fig. 3A). This result indicated that pyroptosis inhibited recovery from SNTM in an IL-1β-independent manner. To further determine which inflammasome is responsible for pyroptosis and PNR inhibition during SNTM, we compared the NLRP3, NLRC4, and AIM2 inflammasome-deficient mice in the SNTM model and found that the GSDMD^−/−^ and NLRP3^−/−^ SNTM mice had better SFIs than the NLRC4^−/−^ and AIM2^−/−^ and WT mice, and the GSDMD^−/−^ mice had a slightly better SFI than the NLRP3^−/−^ mice (Fig. 3B).

Furthermore, we used morphological methods to observe the SNTM bridge; the WT SNTM group demonstrated a fiber gap between the proximal and distant nerve stumps that hampered regenerated axons, while a bridge of reconnected axons was found in the NLRP3^−/−^ SNTM group (Fig. 3C–H). These results indicated that NLRP3 inflammasome activation plays a leading role in GSDMD cleavage, which executes pyroptosis in SNTM.

### GSDMD-induced pyroptosis polarized macrophages toward the M1 (detrimental) but not M2 (beneficial) phenotype and thereby dampened the PNR

**Fig. 4:**
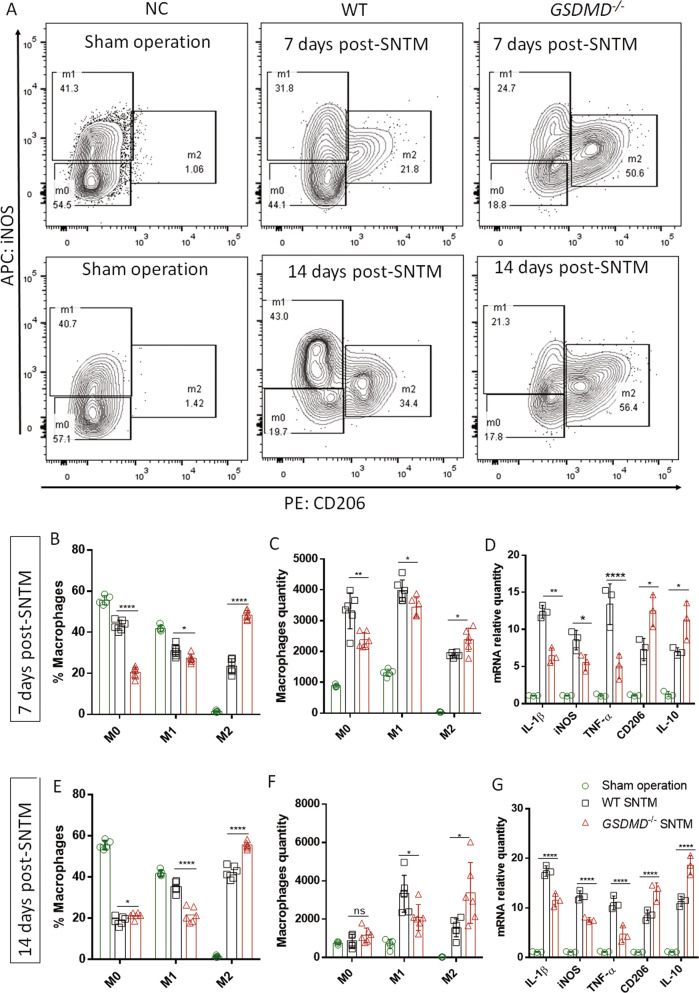
GSDMD-mediated pyroptosis increased M1 polarization while hampering M2 polarization in the SNTM model. **A** Decreased M1 and increased M2 ratios were found on either the 7th or the 14th day post-operation. **B**, **C** The comparison of macrophage phenotypic ratios and quantities, respectively, on the 7th day (NC, *n* = 5; WT or GSDMD^−/−^, *n* = 6). **D** The WT SNTM bridges had higher IL-1β and iNOS and lower CD206 and IL-10 mRNA expression than the *GSDMD*^*−/−*^ SNTM bridges on the 7th day post-operation (*n* = 3). **E**, **F** Comparison of macrophage phenotypic ratios and quantities on the 14th day, respectively (NC, *n* = 5; WT or GSDMD^−/−^, *n* = 6). **G** The WT SNTM bridges had higher IL-1β and iNOS and lower CD206 and IL-10 mRNA expression than those of the *GSDMD*^−*/*−^ group on the 14th day post-operation (*n* = 3). *p < 0.05; **p < 0.01; ***p < 0.001; ****p < 0.0001.

During tissue damage and repair, responsive macrophages and inflammatory monocytes undergo marked functional alterations and phenotypic polarization (M1/M2 paradigm) for clearance of the damage and regeneration [25]. However, the impact of GSDMD-induced pyroptosis on macrophage polarization remains unclear. Therefore, we used flow cytometry to assess macrophages and found that the GSDMD^−/−^ mice with SNTM had decreased M1 and increased M2 cell ratios and quantities compared with their WT counterparts, and GSDMD deficiency inhibited inflammation-promoting M1 and influenced repair-enhancing M2 polarization on the 7th day post-SNTM. Moreover, mRNA expression of inflammatory indicators showed consistent results: the WT SNTM bridge had higher levels of the M1 marker IL-1β, TNF-α, and iNOS and lower M2 marker CD206 and IL-10 mRNA expression than the GSDMD^−/−^ bridge (Fig. 4A–D). Furthermore, similar results were identified on the 14th day (Fig. 4E–G). These results suggested that GSDMD-induced pyroptosis increased inflammation-promoting M1 polarization while hampering repair-enhancing M2 polarization, thereby establishing a less favorable proinflammatory microenvironment for PNR.

**Fig. 5:**
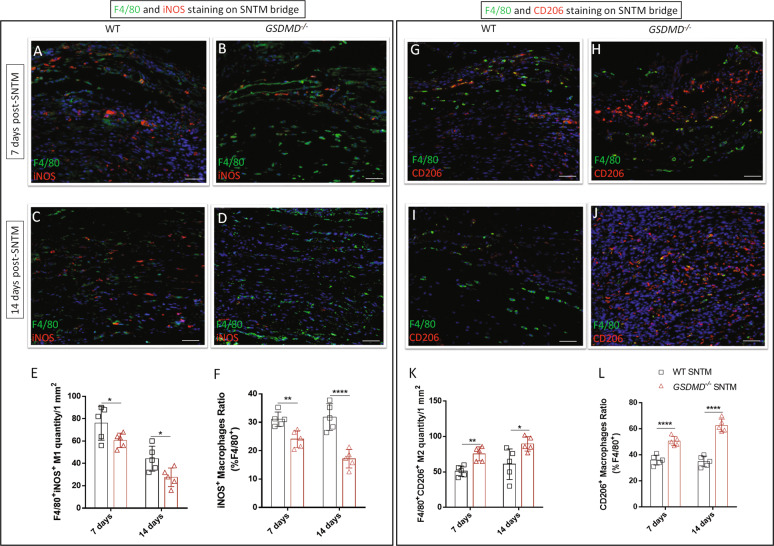
GSDMD^−/−^ SNTM model demonstrated decreased F4/80+iNOS+ M1 macrophages and increased F4/80+CD206+ M2 macrophages in the SNTM bridge. **A**–**D** Comparison of M1 macrophage via F4/80 (green) and iNOS (red) double fluorescent staining on the WT and *GSDMD*^−*/−*^ SNTM on the 7th day and the 14th day, respectively. **E** WT had significantly more F4/80^+^iNOS^+^ M1 macrophages than *GSDMD*^−*/−*^ group in the SNTM bridge (*n* = 5). **F** WT had a significantly higher ratio of iNOS^+^ M1 in F4/80^+^ macrophages than the *GSDMD*^*−/*−^ group in the SNTM bridge (*n* = 5). **G**–**J** Comparison of M2 macrophage via F4/80 (green) and CD206 (red) double fluorescent staining on the WT and *GSDMD*^−*/−*^ SNTM on the 7th day and the 14th day, respectively (*n* = 5). **K** WT had significantly fewer F4/80^+^CD206^+^ M2 macrophages than *GSDMD*^−*/*−^ group in the SNTM bridge. **L** WT had a significantly lower ratio of CD206^+^ M2 in F4/80^+^ macrophages than the *GSDMD*^−*/−*^ group in the SNTM bridge (*n* = 5). *p < 0.05; **p < 0.01; ***p < 0.001; ****p < 0.0001. Scale bar: 50 μm.

Furthermore, we used F4/80 and iNOS as M1 markers, and F4/80 and CD206 as M2 markers for fluorescent IHC staining, in which *GSDMD*^−*/−*^ SNTM had decreased F4/80^+^iNOS^+^ M1 quantities and ratios and increased F4/80^+^CD206^+^M2 cell quantities and ratios compared with their WT counterparts (Fig. 5).

## Discussion

WD involves the recruitment of macrophages for debris clearance and nerve regeneration, and foamy cells constantly appears in peripheral transection injuries, with an unknown cause. A recent discovery indicated that GSDMD-executed pyroptosis can induce the foamy phenomenon in macrophages. However, whether foamy cells during peripheral transection injuries are pyroptotic macrophages and whether cell death elicits immunogenicity in PNR remain unknown. Therefore, we established a SNTM mouse model and found that the GSDMD^−/−^ SNTM group showed a significantly diminished number of foamy cells and parallel axon regeneration and favorable functional recovery, whereas irregular axons or gaps in fibers were found in the WT SNTM group. Furthermore, GSDMD activation in the SNTM was dependent on the NLRP3 inflammasome and caspase-1 activation, and GSDMD-executed pyroptosis established an environment with proinflammatory cytokines that polarized monocytes/macrophages toward the M1 (detrimental) but not M2 (beneficial) phenotype, which amplified the inflammatory cascade that hampered PNR. Therefore, inhibition of GSDMD may be a potential treatment option to promote PNR.

WD can stimulate innate immune responses to induce debris clearance and initiate repair [26], in which cell death-elicited immunogenicity (noninflammatory or proinflammatory) can determine the repair microenvironment and thereby resolve PNR outcomes. In apoptosis, cells undergo programmed cell death in which condensed cells are fragmented into apoptotic bodies that are usually engulfed by surrounding macrophages, resulting in a noninflammatory type of cell death [27]. In contrast, necrosis is a proinflammatory cell death because of the release of intracellular materials [28], and pyroptosis is the other form of programmed cell death, in which lytic cells become swollen and produce large foams. These ruptured foams lead to substantial leakage of cytosolic contents and thereby a cascade of inflammatory reactions [29]. Importantly, the “foamy macrophages near a blood vessel 14 days after transection” phenomenon was identified by G. Stoll in 1989 [12]. These foamy macrophages are pyroptotic macrophages that show GSDMD-N oligomerization and membrane pore formation [10]. Consistently, our study of the WT SNTM model also found these foamy macrophages that undergo pyroptosis, whereas infiltrated macrophages near the blood vessels did not present the foaming phenomenon in the GSDMD^−/−^ SNTM group, which suggested that these macrophages showed alternative cell death patterns (apoptosis, necroptosis, or other types of cell death).

The NLRP3 inflammasome can detect a broad range of DAMPs and pathogen-associated molecular patterns (PAMPs) and activate caspase-1 with the induction of pyroptosis and the release of mature interleukin IL-1β/18 [30], and the inhibition of the NLRP3 inflammasome can reduce inflammatory damage in multiple disease models [31, 32]. In this study, we compared ScN function (SFI) in a series of inflammasome-deficient mouse models that underwent SNTM: the NLRP3^−/−^ mice had a better SFI than the WT, NLRC4^−/−^, and AIM2^−/−^ mice. Although the GSDMD^−/−^ mice had a slightly better functional recovery than the NLRP3^−/−^ mice, our results suggested that GSDMD-induced pyroptosis in PNI was mainly dependent on NLRP3 inflammasome assembly, and whether NLRP3-independent GSDMD activation also dampens the PNR needs to be further investigated.

Mirroring the Th1/Th2 nomenclature, the M1/M2 paradigm refers to “classically activated” proinflammatory, cytotoxic M1 cells (markers of reactive oxygen and nitrogen intermediates, iNOS) with the production of TNF-α, IL-1β, and IL-6 and “alternatively activated” anti-inflammatory, repair-enhancing, tissue-remodeling M2 cells (markers of the scavenger, mannose, and galactose-type receptors) [33]. Generally, Toll-like receptor (TLR) and Nod-like receptor (NLR) activation and cytokine (e.g., TNF-α and IFN-γ) activation can lead to classical M1 polarization, whereas M2 polarization results from glucocorticoid and secosteroid (vitamin D3) hormones and cytokines, including IL-4, IL-10, and IL-13 [4, 34]. Furthermore, in spinal cord injury, macrophages and microglia acquire the M1 phenotype by stimulating TNF-α and increasing intracellular iron [35], and iron (e.g., chelatable redox-active Fe^2+^, Ca^2+^) can stimulate NLRP3 assembly [36, 37]. Overstimulation of M1 macrophages can result in macrophage pyroptosis, which further amplifies cell death-elicited immunogenicity in a forward loop. Consistently, we also found that the macrophages in the WT SNTM group were mainly M1 cells, whereas GSDMD deficiency-induced pyroptosis switched this M1 phenotype to the M2 phenotype to facilitate PNR. Moreover, our study identified increased IL-10 and decreased TNF-α and IL-1β mRNA expression in the GSDMD^−/−^ SNTM group, which indicated that a favorable anti-inflammatory cytokine network was created when GSDMD-induced pyroptosis was inhibited.

In our study, GSDMD depletion can significantly inhibit macrophage pyroptosis and present better PNR function with reduced IL-1β expression, and this result indicated that IL-1β blockade had a potential to benefit PNR; however, the IL-1β^−/−^ SNTM mice did not demonstrate similar SFI outcome compared with GSDMD^−/−^, Capapse-1^−/−^, and NLRP3^−/−^ groups. This result indicated IL-1β performed contradictory functions in determining PNR outcomes, and that contradiction may result from the IL-1β receptor’s extensive distribution in various cell types (i.e., monocytes, epithelium, and endothelial cells) [38] and opposing functions of IL-1β receptor subtypes (IL-1R1 and IL-1R2). Specifically, IL-1R1 can bind adapter molecular myeloid differentiation primary response 88 (Myd88) and activate NF-κB signaling, thereby initiating acute inflammation [39], while IL-1R2 performed as a negative regulator of IL-1R1/Myd88 signaling by competitive inhibition with IL-1R1 for IL-1 [40]. In previous studies, similar contradictory results were reported in the animal model of peripheral nerve diseases and injuries: IL-1β could enhance PNR by promoting Schwann cells de-differentiation in WD [23], and IL-1R antagonist can impede PNR [41]; contrarily, IL-1β signaling blockade can protect peripheral nerves from axonal loss and cell death in a mouse model of familial amyloidotic polyneuropathy [42]. In addition, the pyroptotic released immunogenic materials and the other pyroptotic proinflammatory cytokine IL-18s effects in PNR should also be assessed in further studies.

Many studies have focused on the PNR and its mechanism during different phases of WD. However, a standardized protocol for peripheral nerve transection injury in animal models remains elusive. Furthermore, the M1/M2 paradigm depends on the tissue environment accompanied by WD, in which Schwann cells, macrophages, and various cytokines shift rapidly and dynamically and cannot be precisely assessed by a single method. Whether other types of cells, such as Schwann cells, can also execute pyroptosis needs to be addressed in the future.

## Supplementary information

Supplemental Tables

Supplemental Fig. 1. The GSDMD-/- mouse model of SNTM with the epineurial repair technique.

Supplemental Fig. 2. The gating strategy of macrophage phenotypes.
