# Supplementary material for: Gasdermin D in peripheral nerves: the pyroptotic microenvironment inhibits nerve regeneration
Source: Cell Death Discov. 2021 Jun 14;7:144. doi: 10.1038/s41420-021-00529-6 (PMC8203780; doi:10.1038/s41420-021-00529-6)
Supplement: Supplementary file 1 — Supplemental Tables [file 41420_2021_529_MOESM1_ESM.docx]

**Supplemental Table. 1.**

Lists of primers used for Q-RT-PCR

| Primer | forward | reverse |
| --- | --- | --- |
| Mouse IL-1β | 5'- TGGACCTTCCAGGATGAGGACA-3' | 5'- GTTCATCTCGGAGCCTGTAGTG-3' |
| Mouse iNOS | 5'- CGAAACGCTTCACTTCCAA-3' | 5'- TGAGCCTATATTGCTGTGGCT-3' |
| Mouse TNF-α | 5'-GGTGCCTATGTCTCAGCCTCTT- 3' | 5'- GCCATAGAACTGATGAGAGGGAG -3' |
| Mouse CD206 | 5'-CTCTGTTCAGCTATTGGACGC -3' | 5'- CGGAATTTCTGGGATTCAGCTTC-3' |
| Mouse IL-10 | 5'-CGGGAAGACAATAACTGCACCC-3’ | 5’-CGGTTAGCAGTATGTTGTCCAGC-3’ |
| Mouse GAPDH | 5'-GTCTTCCTGGGCAAGCAGTA-3’ | 5’-CTGGACAGAAACCCCACTTC-3’ |

**Supplemental Table. 2. Animal sample preparation Procedures**

| **Item** | **Time** | **Process** | **Notes** |
| --- | --- | --- | --- |
| **Sample collection** | **1^st^ day** | 1. Prepare clean and sharp knives, scissors, and punches for sampling.  2. Quickly separate and cut tissues, try to avoid pulling, sawing, pressing and other actions.  3. Put the tissue into the fixative solution 3% glutaraldehyde (2% paraformaldehyde) as soon as possible. The amount of fixation solution used should be 1 ml per sample.  4. The size of the sample block does not exceed than1 cubic millimeter | 1. The location of the material must be accurate.  2. Individual differences should be minimized when sampling.  3. The fixed time should not exceed one week. |
| **Double Fixation** | **2^nd^ day** | 1. Take out the embedding agent from the refrigerator and stir at room temperature.  2. Aspirate the glutaraldehyde in the sample and put it into the recovery bottle.  3. Add 0.1M PBS buffer (PH=7.2) about 1ml room temperature and rinse three times, each  Replace every 7 minutes | 1. Try to avoid the sample sticking to the wall, and lightly flick the tube wall.  2. Pay attention to fragile or very small samples. |
|  |  | Aspirate and discard the solution, and fix with 1% osmium tetroxide at room temperature. The amount shall be based on the sample that has not been used. Observe whether the sample is completely darkened after 60 minutes. Fix for a maximum of 90 minutes. | 1. Osmium acid is toxic and volatile, so it should be operated in a fume hood with gloves.  2. There is ethanol in the osmic acid waste liquid recovery bottle, which can react with osmic acid to make it completely precipitate. |
|  |  | Recover the waste liquid, wash it three times with about 1ml of 0.1M PBS buffer (PH=7.2) at room temperature, and replace it every 7 minutes. The cleaning must be thorough, otherwise the osmium will react with the ethanol behind to form a precipitate. Note that the residual liquid in the cap should be treated at the same time when cleaning |  |
| **Dehydration** | **2^nd^ day** | 1. Aspirate and discard the solution, add 1ml of 30% ethanol, at room temperature for 10 minutes.  2. Aspirate and discard the solution, add 1ml of 50% ethanol, at room temperature for 10 minutes.  3. Aspirate and discard the solution, add 1ml of 70% ethanol, at room temperature for 10 minutes.  4. Aspirate and discard the solution, add 1ml of 80% ethanol, at room temperature for 10 minutes.  5. Aspirate and discard the solution, add 1ml of 95% ethanol, at room temperature for 10 minutes.  6. Aspirate and discard the solution, and add 1ml of absolute ethanol at room temperature for 10 minutes.  7. Aspirate and discard the solution, rinse with 1ml of acetone three times, 10 minutes each time, pay attention to recovering acetone.  Throw out the trash once, put in a new trash bag, and spread the new paper. | 1. Dehydration must be thorough.  2. The action must be rapid when changing the liquid, otherwise an air film will be formed on the sample surface, which will affect the effect of dehydration and the next step of infiltration. |
| **Infiltration** | **2^nd^ day** | 1. Absorb the solution, add acetone: epoxy resin = 1:1, the amount is about 500μl, and place it on the rocker for 1 hour at room temperature.  2. Absorb the solution, add acetone: epoxy resin = 3:1, the amount is about 500μl, and place it on the rocker for 3 hours at room temperature.  3. Aspirate and discard the solution, add about 250μl of epoxy resin, and place it on the rocker overnight at room temperature after replacement. | 1. The embedding agent is toxic before polymerization, so it should be operated in a fume hood with gloves. Note that the embedding agent should not get on the experimental equipment, especially the pipette.  2. Pay attention to selecting a suitable embedding plate and clean it up.  3. Be careful not to cover the bottom of the hole with sulphuric acid paper and leave a position for the slice.  4. Take care to avoid air bubbles as much as possible, use a toothpick to check whether there is air hidden under the sulfuric acid paper.  5. Pay attention to check whether the temperature of the oven is 70℃ |
| **Embedding** | **3^rd^ day** | At about 10 am, replace the epoxy resin with a dosage of about 250μl, and still place it on the rocker.  2. At about 3 pm, replace the epoxy resin with an amount of about 250μl and still place it on the rocker.  3. At about 5 pm, fill the embedding tube or embedding plate with epoxy resin, pick up the sample with a toothpick, and put it into the embedding tube or embedding plate, and note the name of each sample, and place it at room temperature. overnight.  4. Pour the remaining embedding agent and sample into the trash bag, put it in the oven together with the newspaper and throw it away after polymerization.  5. Immediately use absolute ethanol to dissolve the remaining embedding agent in the beaker, recover the waste liquid, and then clean the beaker with absolute ethanol, rinse it with distilled water three times, and put it in an oven to dry for later use. |  |
| **Embedding block** | **4^th^ day** | 1. Place the embedding plate on the top layer of a 70°C oven and polymerize for at least 48 hours.  2. Take out the embedding plate, carefully take out the embedding block after cooling, and use a toothpick to remove the residual embedding agent in the hole. |  |
