# Supplementary figures and images for: Gasdermin D in peripheral nerves: the pyroptotic microenvironment inhibits nerve regeneration
Source: Cell Death Discov. 2021 Jun 14;7:144. doi: 10.1038/s41420-021-00529-6 (PMC8203780; doi:10.1038/s41420-021-00529-6)

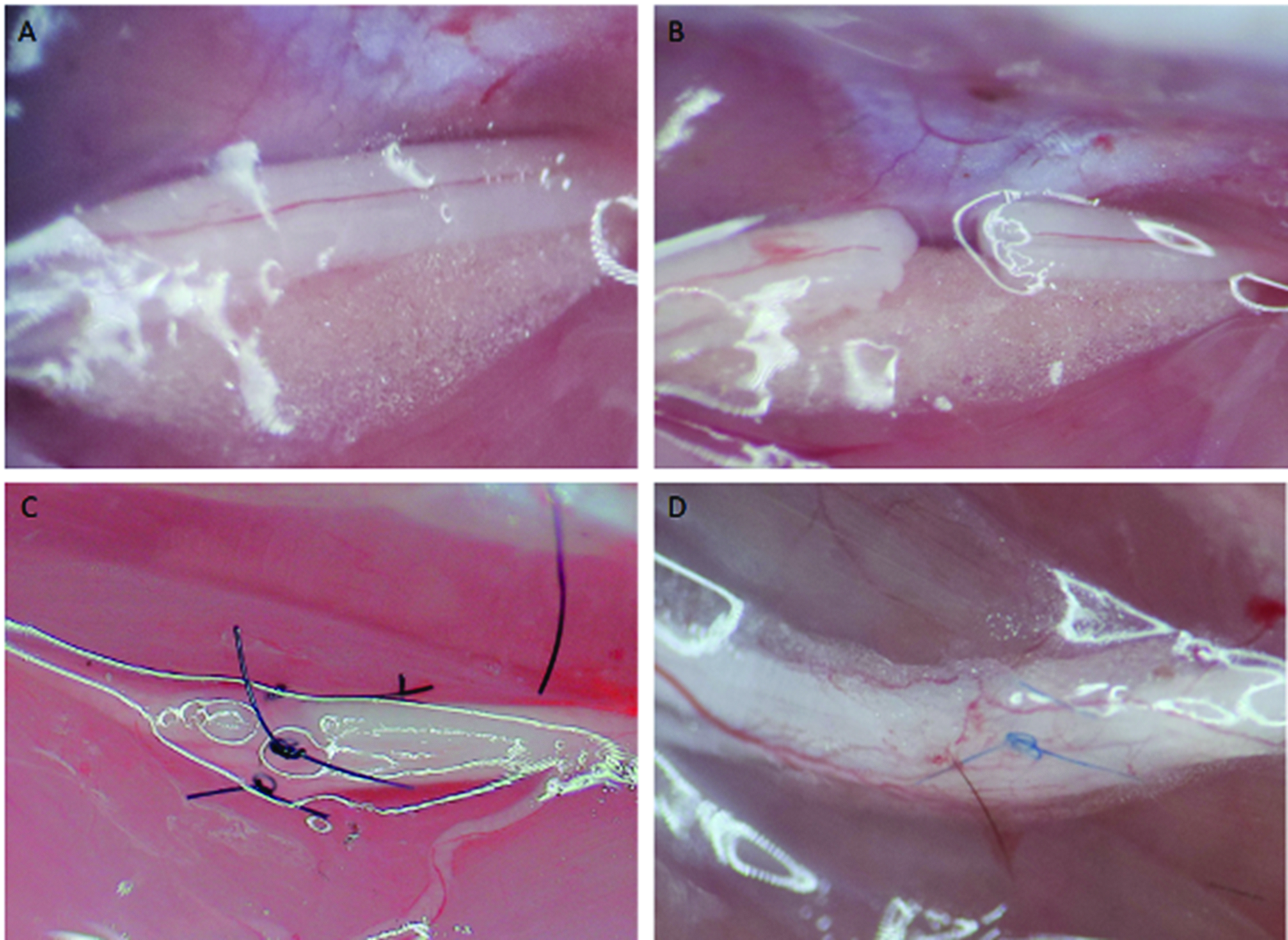

Supplement: Supplementary file 2 — Supplemental Fig. 1. The GSDMD-/- mouse model of SNTM with the epineurial repair technique. [file 41420_2021_529_MOESM2_ESM.tif]
